# Using PyBioNetFit to Leverage Qualitative and Quantitative Data in Biological Model Parameterization and Uncertainty Quantification

**Published:** 2025-08-26

**Authors:** Ely F. Miller, Abhishek Mallela, Jacob Neumann, Yen Ting Lin, William S. Hlavacek, Richard G. Posner

**Affiliations:** 1 Department of Biological Sciences, Northern Arizona University, Flagstaff, AZ, United States of America; 2 Center for Nonlinear Studies, Los Alamos National Laboratory, Los Alamos, NM, United States of America; 3 Theoretical Biology and Biophysics Group, Theoretical Division, Los Alamos National Laboratory, Los Alamos, NM, United States of America; 4 Department of Chemistry and Chemical Biology, Cornell University, Ithaca, NY, United States of America; 5 Information Sciences Group, Computer, Computational and Statistical Sciences Division, Los Alamos National Laboratory, Los Alamos, NM, United States of America

**Keywords:** Curve-fitting, Maximum likelihood estimation (MLE), Profile likelihood, Bayesian inference, Markov chain Monte Carlo (MCMC)

## Abstract

Data generated in studies of cellular regulatory systems are often qualitative. For example, measurements of signaling readouts in the presence and absence of mutations may reveal a rank ordering of responses across conditions but not the precise extents of mutation-induced differences. Qualitative data are often ignored by mathematical modelers or are considered in an *ad hoc* manner, as in the study of Kocieniewski and Lipniacki (2013) [*Phys Biol* **10**: 035006], which was focused on the roles of MEK isoforms in ERK activation. In this earlier study, model parameter values were tuned manually to obtain consistency with a combination of qualitative and quantitative data. This approach is not reproducible, nor does it provide insights into parametric or prediction uncertainties. Here, starting from the same data and the same ordinary differential equation (ODE) model structure, we generate formalized statements of qualitative observations, making these observations more reusable, and we improve the model parameterization procedure by applying a systematic and automated approach enabled by the software package PyBioNetFit. We also demonstrate uncertainty quantification (UQ), which was absent in the original study. Our results show that PyBioNetFit enables qualitative data to be leveraged, together with quantitative data, in parameterization of systems biology models and facilitates UQ. These capabilities are important for reliable estimation of model parameters and model analyses in studies of cellular regulatory systems and reproducibility.

## 1 Introduction

In systems biology modeling, practitioners routinely disregard purely qualitative data—such as ordinal categorizations (e.g., low/medium/high), binary outcomes of case-control comparisons (e.g., up/down relative to a reference), and simple threshold crossing indicators (e.g., yes/no)—as well as semi-quantitative data, such as densitometric readouts of Western blots, which typically lack full quantification due to potential signal saturation and absence of calibration experiments. Bias against use of such data perhaps arises from the rich abundance of quantitative data (e.g., sequential measurements of state variables having high precision and fine time resolution) in fields such as physics, in which biological modelers are commonly trained. Nevertheless, qualitative/semi-quantitative data have been leveraged in influential biological modeling studies (Mitra and Hlavacek, 2019), such as those of Tyson and co-workers focused on modeling yeast cell-cycle control (Chen et al., 2000; Chen et al., 2004; Kraikivski et al., 2015; Barik et al., 2016; Tyson et al., 2019). In these studies, qualitative/narrative descriptions of yeast mutant phenotypes were used to constrain parameter estimates. Other related studies include those of Kinney and co-workers focused on using noisy semi-quantitative readouts of massively parallel reporter assays to inform the modeling of regulation of gene expression (Kinney et al., 2007; 2010; Atwal and Kinney, 2016; Kinney and McCandlish, 2019).

In recent years, there has been increasing appreciation of the need to leverage all types of available data in biological modeling, as evidenced by the development and/or application of various approaches for using qualitative/semi-quantitative data in model parameterization, optionally in combination with quantitative data. Demonstrated approaches include likelihood-free inference conditioned on binary data (Toni et al., 2011), data-transformation methods (e.g., optimal scaling) (Pargett and Umulis, 2013; Pargett et al., 2014; Schmiester et al., 2020; 2021; Dorešić et al., 2024), information-theoretic approaches (Atwal and Kinney, 2016; Tareen et al., 2022), constrained heuristic optimization in which qualitative observations are formalized as constraints (Oguz et al., 2013; Mitra et al., 2018), and (multinomial) probit regression (Mitra and Hlavacek, 2020). Software tools developed to cope with qualitative/semi-quantitative data include PyBioNetFit (Mitra et al., 2019; Mitra and Hlavacek, 2020), pyPESTO (Schmiester et al., 2021; Schälte et al., 2023; Dorešić et al., 2024), and MAVE-NN (Tareen et al., 2022).

Here, in a new demonstration of the ability of PyBioNetFit to leverage both quantitative and qualitative data, we redo the parameterization of an ordinary differential equation (ODE) model developed by Kocieniewski and Lipniacki (2013) to study the roles of MEK isoforms in ERK activation. Originally, this model was parameterized by tedious trial-and-error manual tuning of parameter values for consistency with a mix of published qualitative and quantitative data (Catalanotti et al., 2009; Kamioka et al., 2010). The qualitative data included orderings of low-resolution readouts measured for parental and perturbed/mutant cell lines. In our re-analyses of these data, we define qualitative data unambiguously using the Biological Property Specification Language (BPSL) (Mitra et al., 2019), which increases the reusability of these data, and we leverage both the qualitative and quantitative data using various features of PyBioNetFit, which yields a systematic and automated approach to parameter estimation and uncertainty quantification (UQ). This approach increases reproducibility. Furthermore, we clarify that the parameter estimation method of Mitra et al. (2018), which is implemented in PyBioNetFit (Mitra et al., 2019) and which we leveraged in our study, can be viewed as a likelihood-based inference procedure. Through metaheuristic optimization, we obtain maximum likelihood estimates for parameter values, and for UQ, we perform profile likelihood analysis (Kreutz et al., 2013). In addition, through application of an adaptive Markov chain Monte Carlo (MCMC) sampling algorithm (Andrieu and Thoms, 2008) implemented in PyBioNetFit (Neumann et al., 2022), we obtain samples representing a Bayesian parameter posterior, which allows us to quantify both parametric and prediction uncertainties. Prediction uncertainties are quantified by posterior predictive distributions. Thus, we demonstrate UQ in two ways, each of which considers both quantitative and qualitative data. The absence of UQ was a notable limitation of the original study of Kocieniewski and Lipniacki (2013), a limitation rooted in the *ad hoc* labor-intensive parameterization approach of that study.

The results presented here provide a new illustration of how PyBioNetFit facilitates efficient and reproducible use of qualitative data (together with quantitative data) in parameterization of systems biology models. The results also provide an illustration of PyBioNetFit capabilities useful for UQ, which is essential for a variety of reasons, from assessment of model credibility to model improvement. This report should be useful to researchers who wish to leverage qualitative data in parameterization of biological models.

## 2 Materials and Methods

### 2.1 Model for parental cell line and model variants for mutant cell lines

Kocieniewski and Lipniacki (2013) defined their model for normal, or wild type (WT), MEK1 signaling using the conventions of the BioNetGen language (BNGL) (Faeder et al., 2009) and made the model available online in the form of a BNGL file (a plain-text file with a .bngl filename extension) within a ZIP archive (https://iopscience.iop.org/article/10.1088/1478-3975/10/3/035006/data). Annotation at the top of this BNGL file describes how to modify the model to account for MEK1 knockout (KO) and three MEK1 mutations: N78G, which ablates MEK1 dimerization with itself and MEK2; T292A, which ablates ERK-mediated inhibitory phosphorylation of MEK1 and concomitant negative feedback; and T292D, a phosphomimetic mutation. From this starting point, we created separate BNGL files for parental and mutant cell lines labeled WT, N78G, T292A, and T292D. The resulting BNGL files, MEK1_WT.bngl, MEK1_KO.bngl, MEK1_N78G.bngl, MEK1_T292A.bngl, and MEK1_T292D.bngl, are freely available online (https://github.com/lanl/PyBNF/tree/master/examples/Miller2025_MEK_Isoforms) and can be processed by PyBioNetFit (Mitra et al., 2019), version 1.1.9. In PyBioNetFit workflows, models can be defined using either BNGL or Systems Biology Markup Language (SBML) (Keating et al., 2020). BioNetGen (Harris et al., 2016) can convert a BNGL file to an SBML file. SBML files are plain-text files with .xml filename extensions.

### 2.2 Data used in model parameterization

Kocieniewski and Lipniacki (2013) carefully identified the data that they used in model parameterization. We collected these same data from the primary sources, namely the reports of Catalanotti et al. (2009) and Kamioka et al. (2010). Each qualitative observation from the case-control comparisons of Catanotti et al. (2009)—e.g., up/down relative to a reference at a particular time after initiation of signaling—was formalized using the Biological Property Specification Language (BPSL) (Mitra et al., 2019). BPSL statements, which can be interpreted by PyBioNetFit, were then collected in PROP files (plain-text files with .prop filename extensions). See BPSL examples and breakdowns in the box below. Quantitative observations, time-series data, from the study of Kamioka et al. (2010) were collected/tabulated in an EXP file (a plain-text file with a .exp filename extension). It should be noted that, in an EXP file, “nan” indicates a missing measurement. The resulting EXP and PROP files are freely available online (https://github.com/lanl/PyBNF/tree/master/examples/Miller2025_MEK_Isoforms) and can be processed by PyBioNetFit (Mitra et al., 2019), version 1.1.9. It should be noted that each EXP and PROP file is meant to be used with a particular model variant, as indicated by the labels WT, N78G, T292A, and T292D. For example, during model parameterization, the data collected in the WT-labeled EXP and PROP files (WT.exp and WT.prop) are compared against the corresponding outputs of the WT model (defined in the MEK1_WT.bngl file). The relevant model outputs are identified in accordance with established conventions (Mitra et al., 2019); for example, in an EXP file, times are indicated by row labels and each column header has the name of an observable or function defined in a BNGL file. We note that the only non-empty EXP file maps to the WT model, because quantitative, time-series data was only available for normal signaling (i.e., signaling unperturbed by MEK1 knockout or mutation).

**Table:** 

BPSL Statement	Prose Statement
WT.MEK_pRDS at time = 300 < N78G. MEK_pRDS at time = 300	WT MEK copy number at 300 seconds is less than N78G MEK copy number at 300 seconds.
KO.MEK_pRDS at time = 300 > KO.MEK_pRDS at time = 1800	KO MEK copy number at 300 seconds is greater than KO MEK copy number at 1800 seconds.
N78G. MEK_pRDS at time = 300 > T292D.MEK_pRDS at time = 300	N78G MEK copy number at 300 seconds is greater than T292D MEK copy number at 300 seconds.

### 2.3 Maximum likelihood estimation and likelihood profiling

Across the five model variants under consideration (WT, KO, N78G, T292A, and T292D), there are 46 total parameters. We took 28 of these to be adjustable and set the other six at fixed values specified by Kocieniewski and Lipniacki (2013). Six of these parameters $\left(s_{1},d_{1},s_{2},d_{2},c_{1},c1_{\mathrm{init}}\right)$ were fixed due to added computational complexity to our simulations. In the model by Kocieniewski and Lipniacki (2013), the six parameters are responsible for generating the initial levels of EGFR, Sos1, and EGFR/ligand subcomplexes necessary to complete the full MEK isoform cascade. Instead of using the “setup” simulation, we initially simulated the model using these parameters, captured the peak copy number of each subunit, and used peak copy numbers to start off the full cascade in the model rather than using the initial “setup”. This preserved the starting conditions for the model while saving runtime in our simulations. These specific fixed parameter settings can be found in the BNGL files that define the five models.

The remaining 12 fixed parameters (X, $MEK_{-}Fraction$, $EGFR_{0}$, $SOS1_{0}$, $RAS_{0}$, $RAF_{0}$, $MEK_{{\mathrm{tot}}_{0}}$, $MEK1_{0}$, $MEK2_{0}$, $MEK1_{-}T292p_{0}$, $ERK_{0}$, $PHP\_MEK_{0}$ are parameters associated with setting up the initial conditions of the cell in a resting cell steady state. The other six fixed parameters, discussed above, simulated the initial copy numbers of $EGFR_{0}$ and $SOS1_{0}$, and gave the starting signal to start the cell signaling cascade. Therefore, in our study, $EGFR_{0}$ and $SOS1_{0}$, were initially simulated using the six parameters above, then fixed by identifying the peak (initial) copy number for each species during the cell setup simulation. The remaining parameters associated with setting up the initial conditions of the cell were fixed by Kocieniewski and Lipniacki (2013) and remained fixed by us when parameterizing the models with PyBioNetFit.

Furthermore, all 28 adjustable parameters chosen to be used in our study were initially centered on Kocieniewski and Lipniacki’s (2013) parameter values and given bounds of one or two orders of magnitude (10x - 100x) in either direction of the initial value. Some parameter bounds were further adjusted if the algorithm began pushing the limit of a given bound. All parameter bounds can be seen in Table 2. In addition, we introduced 3 adjustable scaling factors, which are useful for comparing WT model outputs to serial measurements. Consequently, in parameter estimation, we considered a total of 31 adjustable parameters, which we will denote as θ. To obtain point estimates, $\overset{ˆ}{\theta }$, for the 31 adjustable parameters θ, we minimized an objective function $F(\theta )=F_{\text{qual}}(\theta )+F_{\text{quant}}(\theta )$, the form of which was previously described by Mitra et al. (2018). The objective function accounts for all qualitative data (a total of 90 BPSL statements) and all quantitative data (a total of 18 serial measurements). We constrained $\overset{ˆ}{\theta }$ to lie within a feasible region of parameter space, which we will denote as Θ. The feasible region Θ was defined by box constraints (lower and upper bounds) applied to each adjustable parameter. In summary, we found

(1) $$\overset{ˆ}{\theta }=arg\;\underset{\theta \in \Theta }{min}\;F(\theta )$$

Thus, $\overset{ˆ}{\theta }$ is the product of a global fit. As explained below, the objective function F(θ) is related to a likelihood, and moreover, minimizing the objective function maximizes this likelihood. Thus, our point estimates are maximum likelihood estimates (MLEs).

The fitting problem setup, including identification of each adjustable parameter and the corresponding box constraints, and workflow were defined with a PyBioNetFit configuration, or CONF, file. This file (a plain-text file with a .conf filename extension) is freely available online (https://github.com/lanl/PyBNF/tree/master/examples/Miller2025_MEK_Isoforms/MEK_isoform_optimization_DE). In parameter estimation, the objective function F(θ) was minimized using PyBioNetFit’s implementation of a parallelized differential evolution (DE) algorithm (Mitra et al., 2019). PyBioNetFit-enabled fitting runs were executed within an institutional high-performance computing environment, on the Monsoon cluster at Northern Arizona University. We used 25 of 28 CPUs on a single node within the cluster and the average wall-clock time for optimization simulations were approximately 2 minutes and conducted 2,825 objective function evaluations. The model of CPUs used are Intel(R) Xeon(R) CPU E5–2680 v4 CPUs. Multiple runs were performed, with each run starting at a randomly chosen point within the feasible region of parameter space Θ. The CONF file includes algorithmic parameter settings (e.g., population size) and maps EXP and PROP files to model variants.

Profile likelihood analysis (Kreutz et al., 2013) was performed as described by Mitra et al. (2018). Computation of a profile involves repeatedly solving the parameterization problem described above, but with one selected parameter in θ held fixed at a specified value, which is varied across the profile.

### 2.4 Bayesian inference and uncertainty quantification

In Bayesian inference and uncertainty quantification, for simplicity, we considered only two adjustable model parameters: $d_{3}$, a rate constant characterizing degradation of ligand-bound epidermal growth factor receptor (EGFR) dimers, and $u_{3}$, a rate constant characterizing phosphatase activity responsible for reversal of ERK-mediated negative-feedback phosphorylation of SOS1. Both parameters were deemed practically identifiable by profile likelihood. The adjustable parameters also included three scaling factors, as in fitting. Finally, the adjustable parameters included a noise model parameter, σ. In Bayesian inference, we used a likelihood function described by Mitra and Hlavacek (2020), which has σ as a hyperparameter, and a proper uniform prior defined by box constraints on each of the six adjustable parameters (see below).

Bayesian inference was enabled by the adaptive Markov chain Monte Carlo (MCMC) sampler implemented in PyBioNetFit (Neumann et al., 2022), version 1.1.9. The sampling problem setup and workflow were defined with a PyBioNetFit configuration, or CONF file. This file (a plain-text file with a .conf filename extension) is freely available online (https://github.com/lanl/PyBNF/tree/master/examples/Miller2025_MEK_Isoforms/MEK_isoform_aMCMC). The CONF file specifies the box constraints that define the prior. Each sampling job included 25,000 burn-in iterations, 25,000 adaptation iterations (used to tune the covariance matrix of the proposal kernel), and 250,000 production iterations. We generated five independent chains. Each chain was initialized at a random point within the prior. All five chains converged to the same posterior distribution. Convergence was evaluated by inspecting diagnostic trace plots and pairs plots. We also calculated convergence metrics (Vehtari et al., 2021) using the rstan R package. These metrics indicated convergence according to the guidance of Vehtari et al. (2021). PyBioNetFit-enabled sampling jobs were executed within an institutional high-performance computing environment, on the Monsoon cluster at Northern Arizona University. In the Monsoon cluster at Northern Arizona University, we used 25 CPUs of a single 28 CPU node containing Intel(R) Xeon(R) CPU E5–2680 v4 CPUs, and the approximate wall-clock time for our Bayesian inference simulations were 5.15 days, having conducted 1.25 million objective function evaluations.

### 2.5 Derivation of likelihood function

In this study, we consider a set of model variants ℳ and a dataset 𝒟={y,z}. The dataset consists of n quantitative observations, $y=\left\{y_{1},\ldots ,y_{n}\right\}$, and m qualitative observations, $z=\left\{z_{n+1},\ldots ,z_{n+m}\right\}$. As a simplification, we assume that the n+m obpervations are independent.

The quantitative observations y are relative intensity measurements, which have been scaled such that $y_{i}\in (0,1]$ for i=1,…,n. There are no replicate measurements, and each $y_{i}$ corresponds to a unique measurement condition, $c_{i}$. For each experimental readout $y_{i}$, there is a corresponding model output $f\left(c_{i},\theta \right)$, which depends on condition $c_{i}$ and P adjustable parameters, $\theta =\left\{\theta_{1},\ldots ,\theta_{P}\right\}$. The model output $f\left(c_{i},\theta \right)$ is generated by the model variant in ℳ matched to condition $c_{i}$, which is always the WT model (because time-series data are not available for any of the mutant cells).

Each qualitative observation $z_{i}\in \{0,1\}(i=n+1,\ldots ,n+m)$ is the binary outcome of a comparison of two semi-quantitative measurements ${𝒜}_{𝒾}$ and ${ℬ}_{𝒾}$ made for two different cell lines (e.g., parental and mutant cells) or at two different time points within the same cellular background. By convention, we take $z_{i}=0$ to indicate ${𝒜}_{𝒾}<{ℬ}_{𝒾}$ and $z_{i}=1$ to indicate ${𝒜}_{𝒾}\geq {ℬ}_{𝒾}$. The measurements ${𝒜}_{𝒾}$ and ${ℬ}_{𝒾}$ are made at conditions $a_{i}$ and $b_{i}$, which are identical except for the difference in cell type or time. For each pair of measurements $\left({𝒜}_{𝒾},{ℬ}_{𝒾}\right)$, there is a pair of model outputs $\left(g\left(a_{i},\theta \right),g\left(b_{i},\theta \right)\right)$. The output $g\left(a_{i},\theta \right)$ is generated by the model variant in ℳ matched to condition $a_{i}$, which encompasses cell type or time, and similarly, the output $g\left(b_{i},\theta \right)$ is generated by the model variant matched to $b_{i}$. Moreover, for each $z_{i}$, we have a corresponding model prediction $H\left(g\left(a_{i},\theta \right)-\right.\left.g\left(b_{i},\theta \right)\right)$, where H is the Heaviside function.

In maximum likelihood estimation, we want to find $\overset{ˆ}{\theta }={argmax}_{\theta \in \Theta }ℒ(\theta |𝒟)$, where the likelihood ℒ(θ∣𝒟)=P(𝒟∣θ) is the probability density over the data 𝒟 given model structure and parameter settings. In inference, we view ℒ(θ∣𝒟) as a function of the adjustable parameters θ and take ℒ(θ∣𝒟) to express how probable the data are for given parameter settings. If observations are independent, ℒ(θ∣𝒟)=ℒ(θ∣{y,z}) decomposes into the product ℒ(θ∣y)ℒ(θ∣z), where ℒ(θ∣y) is the likelihood of the quantitative data and ℒ(θ∣z) is the likelihood of the qualitative data.

Let us use $Y_{i}$ to denote a continuous random variable representing the distribution of possible quantitative measurement outcomes of an experiment performed at condition $c_{i}$. We take the observation $y_{i}$ to be a realization of $Y_{i}$, and we take the model output $f\left(c_{i},\theta \right)$ to be a prediction of the expected measurement outcome at condition $c_{i}$. Formally, we take $f\left(c_{i},\theta \right)$ to model $E\left[Y_{i}\right]$. Let us assume normally distributed measurement noise: $Y_{i}∼N\left(f\left(c_{i},\theta \right),\sigma_{i}^{2}\right)$. It then follows that

(2) $$P\left(Y_{i}=y_{i}|f\left(c_{i},\;\theta \right)\right)=\frac{1}{\sigma_{i}\sqrt{2\pi }}exp\left[-\frac{1}{2}{\left(\frac{y_{i}-f\left(c_{i},\;\theta \right)}{\sigma_{i}}\right)}^{2}\right]$$

If the quantitative data $y={\left\{y_{i}\right\}}_{i=1}^{n}$ are taken to be independent, we find

(3) $$P\left(y|{\left\{f\left(c_{i},\theta \right)\right\}}_{i=1}^{n}\right)=\prod_{i=1}^{n}\frac{1}{\sigma_{i}\sqrt{2\pi }}exp\left[-\frac{1}{2}{\left(\frac{y_{i}\;-\;f\left(c_{i},\;\theta \right)}{\sigma_{i}}\right)}^{2}\right]$$

We can identify $P\left(y|{\left\{f\left(c_{i},\theta \right)\right\}}_{i=1}^{n}\right)$ as ℒ(θ∣y). In the absence of information about variances, let us assume that $\sigma_{i}^{2}=\sigma^{2}>0$ for i=1,…,n. Under this assumption of homoscedasticity (i.e., equal variances), we find

(4) $$-lnℒ(\theta |y)=\frac{n}{2}ln\left(2\pi \sigma^{2}\right)+\frac{1}{2\sigma^{2}}\sum_{i=1}^{n}{\left(y_{i}-f\left(c_{i},\theta \right)\right)}^{2}$$

Furthermore,

(5) $$-ln\;ℒ(\theta |y)\propto F_{\text{quant}}\equiv \sum_{i=1}^{n}{\left(y_{i}-f\left(c_{i},\theta \right)\right)}^{2}$$

Note that ℒ(θ∣y) is maximized if we minimize $F_{\text{quant}}$. The expression for $F_{\text{quant}}$ is equivalent to Equation (2) in Mitra et al. (2018).

Let us use $Z_{i},i\in \{n+1,\ldots ,m+n\}$, to denote a discrete random variable representing the distribution of possible outcomes from a comparison of two semi-quantitative experimental readouts ${𝒜}_{𝒾}$ and ${ℬ}_{𝒾}$ made at conditions $a_{i}$ and $b_{i}$. There are only two possible outcomes, which are mutually exclusive: $Z_{i}=0$ (indicating that ${𝒜}_{𝒾}<{ℬ}_{𝒾}$) or $Z_{i}=1$ (indicating that ${𝒜}_{𝒾}\geq {ℬ}_{𝒾}$). We take $z_{i}$ to be a realization of $Z_{i}$. We will assume the following noise model: $Z_{i}∼Bernoulli\left(p_{i}\right)$. Equivalently,

(6) $$Pr\left(Z_{i}=0\right)=1-p_{i}\;\text{and}\;Pr\left(Z_{i}=1\right)=p_{i}$$

Note that $p_{i}$ is the expectation $E\left[z_{i}\right]$. Recall that the model prediction of $z_{i}$ is $H\left(g\left(a_{i},\theta \right)-g\left(b_{i},\theta \right)\right)$. As in standard logistic regression, we will assume that the parameter $p_{i}$ is a sigmoid function of the difference $\delta_{i}\equiv g\left(a_{i},\theta \right)-g\left(b_{i},\theta \right)$:

(7) $$p_{i}=\frac{1}{1\;+\;exp\left[-\delta_{i}/s_{i}\right]}$$

where $s_{i}>0$ is a scale parameter. With this approach, we are assuming that the difference $\delta_{i}$ explains the probability $p_{i}$. If $\delta_{i}\geq 0$, then $p_{i}$ lies between 0.5 and 1 and $z_{i}=1$ is more likely than $z_{i}=0$. Conversely, if $\delta_{i}<0$, then $p_{i}$ lies between 0 and 0.5 and $z_{i}=1$ is less likely than $z_{i}=0$. From the above considerations, we find

(8) $$P\left(Z_{i}=z_{i}|\delta_{i},s_{i}\right)={\left(\frac{1}{1\;+\;e^{-\delta_{i}/s_{i}}}\right)}^{z_{i}}{\left(\frac{e^{-\delta_{i}/s_{i}}}{1\;+\;e^{-\delta_{i}/s_{i}}}\right)}^{1-z_{i}}$$

If the data $z={\left\{z_{i}\right\}}_{i=n+1}^{n+m}$ are independent, we find

(9) $$P\left(z|{\left\{\delta_{i}\right\}}_{i=n+1}^{n+m},{\left\{s_{i}\right\}}_{i=n+1}^{n+m}\right)=\prod_{i=n+1}^{n+m}{\left(\frac{1}{1\;+\;e^{-\delta_{i}/s_{i}}}\right)}^{z_{i}}{\left(\frac{e^{-\delta_{i}/s_{i}}}{1\;+\;e^{-\delta_{i}/s_{i}}}\right)}^{1-z_{i}}$$

We can identify $P\left(z|{\left\{\delta_{i}\right\}}_{l=n+1}^{n+m},{\left\{s_{i}\right\}}_{l=n+1}^{n+m}\right)$ as ℒ(θ∣z). After simplifications, we find

(10) $$-ln\;ℒ(\theta |z)=\sum_{i=n+1}^{n+m}\left[ln\left(1+e^{-\delta_{i}/s_{i}}\right)+\left(1-z_{i}\right)\delta_{i}/s_{i}\right]$$

Furthermore, under an assumption that $\left|\frac{\delta_{i}}{s_{i}}\right|\gg 1$ for i=n+1,…,n+m, we find

(11) $$-ln\;ℒ(\theta |z)\approx F_{\text{qual}}\equiv \sum_{i=n+1}^{n+m}w_{i}\left[max\left(0,-\delta_{i}\right)+\left(1-z_{i}\right)\delta_{i}\right]$$

where $w_{i}=1/s_{i}$. The ith term in the sum can be viewed as a static penalty function with weight $w_{i}$. The penalty is $w_{i}\cdot \left|\delta_{i}\right|$ if the explanatory variable $\delta_{i}$ is inconsistent with the observation $z_{i}$ and 0 otherwise. In the absence of information indicating how categorizations vary over a range of values for the explanatory variable, the weights can be set heuristically as described by Mitra et al. (2018). The above expression for $F_{\text{qual}}$ is equivalent to Equation (3) in Mitra et al. (2018).

In Equation (1), F(θ) is equal to $F_{\text{quant}}(\theta )+F_{\text{qual}}(\theta )$, where $F_{\text{quant}}(\theta )$ is given by Equation (5) and $F_{\text{qual}}(\theta )$ is given by Equation (11).

## 3 Results

Kocieniewski and Lipniacki (2013) developed a collection of related ordinary differential equation (ODE) models for ERK activation dynamics. These models capture and explain MEK isoform-specific effects in cell lines in which MEK1 is normally expressed (WT), knocked out (KO), and mutated at single amino-acid residues (N78G, T292A, and T292D). The models reproduce experimentally characterized signaling behavior, including various qualitative system properties; however, their parameter values were determined through an *ad hoc* manual trial-and-error procedure, precluding straightforward reuse of the qualitative data (up/down assays relative to a reference), reproducibility of the model parameterization approach, and any formal assessment of parametric and prediction uncertainty.

**Figure 1:**
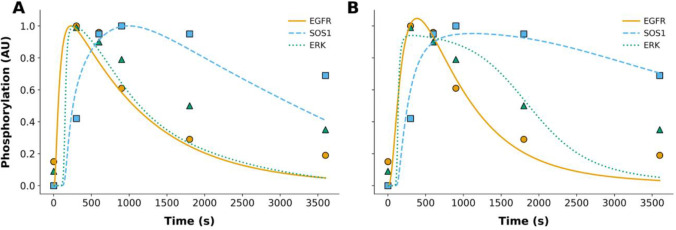
Comparison of model-generated best-fit trajectories with experimental phosphorylation data in WT conditions. Each panel displays simulated trajectories for phosphorylated EGFR (solid orange), SOS1 (dashed light blue), and ERK (dotted green), plotted alongside their respective experimental data points (colored to match model predictions). All trajectories represent Maximum Likelihood Estimates (MLEs). Panel A shows the model outputs using the original parameterization provided by the model’s authors. Panel B presents the MLEs obtained using PyBioNetFit’s differential evolution algorithm with a sum-of-squares objective function. Phosphorylation values are in arbitrary units (AU). Experimental data points are consistent between panels.

**Figure 2:**
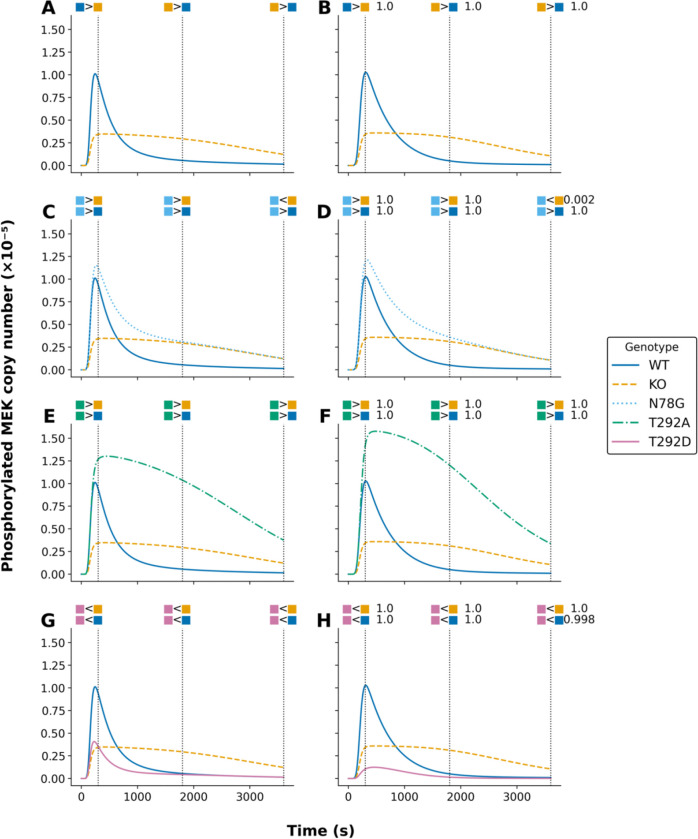
Comparison of model-predicted phosphorylated MEK trajectories under different parameterizations for five model variants. Each subplot illustrates the maximum likelihood estimates (MLEs) of phosphorylated MEK (in molecules ×10^−5^) for a given model. Curves are color-coded by model variant: wild type (WT, solid blue), knockout (KO, dashed orange), N78G (dotted light blue), T292A (dashed-dotted green), and T292D (solid pink). The left panels (A, C, E, G) display model outputs using the original parameters from the models’ authors. The right panels (B, D, F, H) show outputs following re-parameterization via PyBioNetFit using the Differential Evolution (DE) algorithm and a Sum-of-Squares (SOS) objective function. Constraints used in parameterization are represented as colored glyphs above each panel and match the corresponding model variant by color. Vertical black dotted lines indicate the time points at which these constraints apply. Specifically, 300, 1800, and 3600 seconds respectively. For panels B, D, F, and H (PyBioNetFit parameterization), numerical annotations denote the fraction of accepted aMCMC samples satisfying each qualitative constraint. This information is not shown for the original parameterizations (left panels), as they were not derived using Bayesian uncertainty quantification or aMCMC sampling.

**Figure 3:**
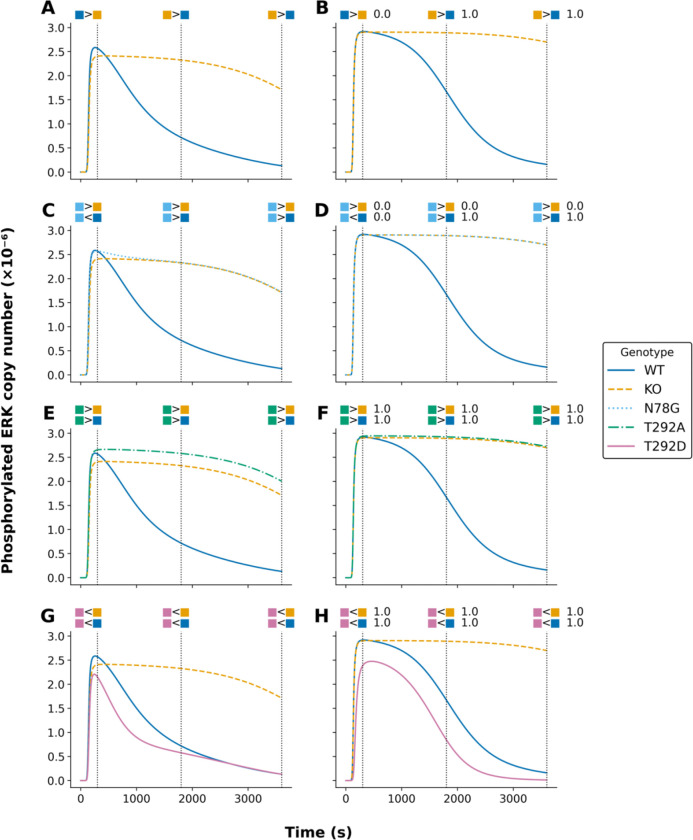
Comparison of model-predicted phosphorylated ERK trajectories under different parameterizations for five model variants. Each subplot illustrates the maximum likelihood estimates (MLEs) of phosphorylated ERK (in molecules ×10^−6^) for a given model. Curves are color-coded by model variant: wild type (WT, solid blue), knockout (KO, dashed orange), N78G (dotted light blue), T292A (dashed-dotted green), and T292D (solid pink). The left panels (A, C, E, G) display model outputs using the original parameters from the models’ authors. The right panels (B, D, F, H) show outputs following re-parameterization via PyBioNetFit using the Differential Evolution (DE) algorithm and a Sum-of-Squares (SOS) objective function. Constraints used in parameterization are represented as colored glyphs above each panel and match the corresponding model variant by color. Vertical black dotted lines indicate the time points at which these constraints apply. Specifically, 300, 1800, and 3600 seconds respectively. For panels B, D, F, and H (PyBioNetFit parameterization), numerical annotations denote the fraction of accepted aMCMC samples satisfying each qualitative constraint. This information is not shown for the original parameterizations (left panels), as they were not derived using Bayesian uncertainty quantification or aMCMC sampling.

To demonstrate a better approach to model parameterization, we formalized qualitative system properties as Biological Property Specification Language (BPSL) statements (Mitra et al., 2019) (Supplemental Tables 1–5). Then, following the inference approach of Mitra et al. (2018), which is elaborated above, we applied, in a single global optimization, a parallelized metaheuristic optimization method implemented in PyBioNetFit (Mitra et al., 2019) to find maximum likelihood estimates (MLEs) for 28 model parameters and 3 scaling factors that relate model outputs to relative measurements (Table 2). The quality of fit to quantitative time-series data (relative measurements) from Kamioka et al., (2010) is illustrated in Figure 1. The quality of fit to qualitative data—up/down assays relative to a reference—from Catalanotti et al., (2009) is illustrated in Figures 2 and 3.

In Figure 1, the left panel (Figure 1A) shows the original fit of Kocieniewski and Lipniacki (2013) to time-series data. The right panel (Figure 1B) shows the comparable fit found in this study. Arguably, the new fit exhibits less systematic error. Note that the model outputs underlying the relative quantities plotted in Figure 1 are the absolute cellular abundances of phosphorylated EGFR, SOS1, and ERK. These quantities are each multiplied by an adjustable scaling factor to align with the relative measurements reported by Kamioka et al. (2010).

The curves in Figures 2 and 3 show calibrated model-predicted phosphorylated MEK and ERK, respectively, as a function of time in different cell lines, as indicated by color and pattern. At the time points corresponding to dotted vertical lines, readouts in pairs of distinct cell lines were compared, resulting in up or down scoring (Catalanotti et al., 2009). The empirical outcomes of these comparisons are indicated graphically above the dotted vertical lines. As in Figure 1, the panels at left (panels A, C, E, and G in both Figures 2 and 3) show the simulation results of Kocieniewski and Lipniacki (2013), and the panels at right (panels B, D, F, and H in both Figures 2 and 3) show the simulation results of this study. As can be seen, we obtained comparable consistency with empirical up/down scoring. Indeed, the calibrated model-predicted time-courses from this study and the earlier study are remarkably similar, despite differences in parameter estimates. The estimates given in Table 2 are far from identical to those given by Kocieniewski and Lipniacki (2013).

We note that Figures 2 and 3 only consider comparisons of readouts in different cell lines. Additional qualitative data were considered in fitting. These data were generated from comparisons of readouts at different time points within the same cellular background. Supplementary Tables 1–5 provide a full listing of the qualitative observations, formalized as BPSL statements, used in fitting.

**Figure 4:**
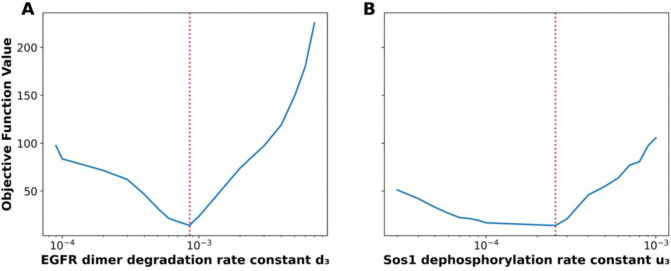
Profile likelihood analysis of two model parameters following PyBioNetFit optimization. This figure shows the results of a profile likelihood analysis performed on the original model to identify parameters with significant influence on the fit to data, aiding selection for adaptive Markov Chain Monte Carlo (aMCMC) sampling. Panel A displays the profile likelihood for the EGFR dimer degradation rate constant $d_{3}$, and Panel B for the Sos1 dephosphorylation rate constant $u_{3}$. Each solid blue curve shows the objective function value (y-axis) when the given parameter (x-axis) is fixed at specific values and the remaining parameters are re-optimized. The vertical red dashed line in each panel indicates the location of the global minimum, the Maximum Likelihood Estimate (MLE), obtained during differential evolution optimization. The curvature and steepness of the profiles suggest sensitivity of the model’s fit to these parameters.

Given the discrepancy between the parameter estimates found here and those of the original study, questions of parametric uncertainty naturally arise. To illustrate that our approach allows for uncertainty quantification (UQ), we generated profile likelihood plots for two rate constants in the models, $d_{3}$ and $u_{3}$ (Figure 4). These plots show that both parameters are practically identifiable and that estimation of the value for $d_{3}$ is more constrained by the available data than is estimation of the value for $u_{3}$. In other words, the width of any horizontal line segment interior to the profile of Figure 4A (solid blue curve) is shorter than the corresponding line segment in Figure 4B. Note that the dotted red vertical line in each panel marks the best-fit estimate.

**Figure 5:**
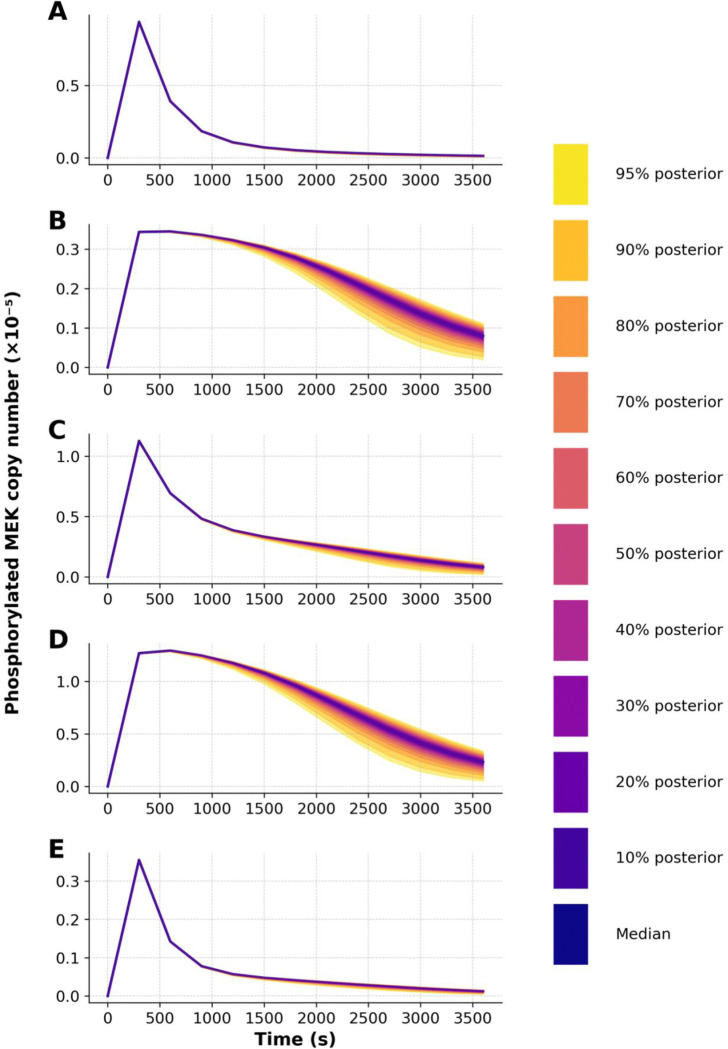
Posterior predictive distributions of phosphorylated MEK copy number for each of the five models, generated using PyBioNetFit’s adaptive Markov Chain Monte Carlo (aMCMC) algorithm with a Chi-Squared Dynamic objective function. Each panel represents one model: **(A)** Wild Type (WT), **(B)** KO, **(C)** N78G, (**D)** T292A, and **(E)** T292D. The dark blue line represents the posterior median prediction, and shaded bands denote posterior credible intervals from 10% to 95%, with the outermost band reflecting the 95% credible interval (CI). These predictions were obtained by fitting to qualitative constraints defined via the Biological Property Specification Language (BPSL), without incorporating measurement noise. The absence of noise leads to narrower posterior uncertainty in some models, particularly Panel A. This figure highlights the degree of confidence in each model’s predictions of MEK phosphorylation dynamics over time, as inferred from posterior parameter distributions.

**Figure 6:**
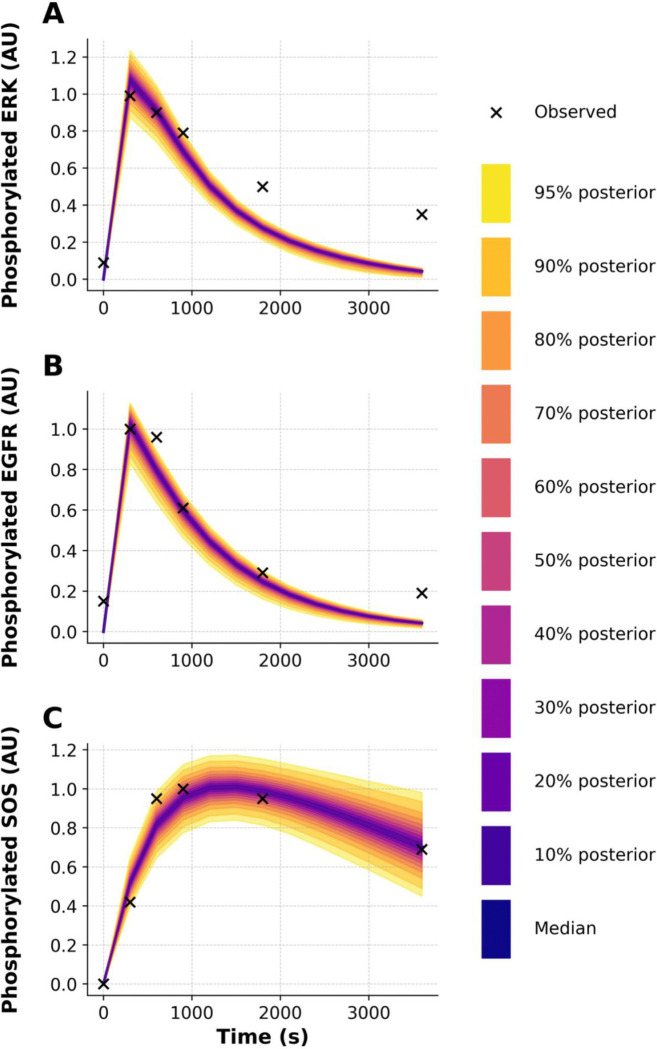
Posterior predictive distributions of phosphorylated signaling proteins from the WT model, computed using adaptive Markov Chain Monte Carlo (aMCMC) sampling in PyBioNetFit with the Chi-Squared Dynamic objective function. Each panel compares the posterior model predictions to the original experimental data used during optimization (aided by qualitative constraints in BPSL): **(A)** phosphorylated ERK, **(B)** phosphorylated EGFR, and **(C)** phosphorylated SOS1, all expressed in arbitrary units (AU). Dark blue lines represent the posterior median trajectories, and colored bands indicate credible intervals from 10% to 95%, with the outermost band corresponding to the 95% credible interval (CI). Although the data points are included in each panel, some fall outside the predicted 95% CI due to the absence of injected measurement noise during sampling.

**Figure 7:**
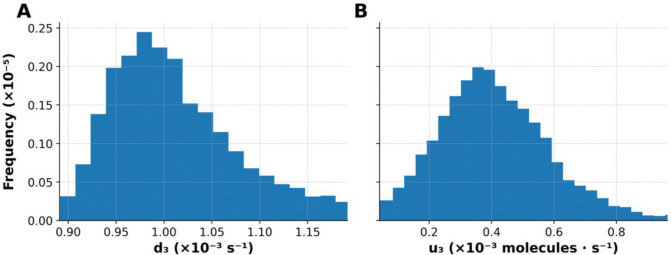
Marginal posterior distributions for the two sensitive parameters used in adaptive MCMC sampling of the 5 models. Panel (A) displays the marginal posterior distribution for the EGFR dimer degradation rate constant $d_{3}$, and panel (B) shows the posterior distribution for the Sos1 dephosphorylation rate constant $u_{3}$. These histograms were generated from the adaptive MCMC samples used in Figures 5 and 6 and illustrate the uncertainty in parameter estimates informed by model optimization and qualitative constraint fitting via PyBioNetFit using the chi-squared dynamic objective function. The shapes of the distributions reflect both parameter identifiability and the lack of injected measurement error in the data, which affects the width and modality of

To further demonstrate the capabilities of PyBioNetFit, we executed a Bayesian approach to inference and UQ, which leveraged the same datasets as those considered in maximum likelihood estimation. However, for the sake of simplicity, we focused on a smaller set of adjustable parameters. Through Markov chain Monte Carlo (MCMC) sampling, we were able to obtain samples converging on and representing the parameter posterior distribution. Sampling convergence metrics are reported in Table 3. Additional convergence diagnostics, likelihood and parameter trace plots and pairs plots, are shown in Supplementary Figures 1–3. Marginal posterior distributions for the rate constants $d_{3}$ and $u_{3}$ are shown in Figure 5. By propagating parametric uncertainty through simulations, we obtained posterior predictive distributions characterizing uncertainty in model predictions (Figures 6 and 7). Supplementary Table 6 quantifies consistency with qualitative observations.

## 4 Discussion

In this study, we demonstrated that PyBioNetFit (Mitra et al., 2019) can replace manual trial- and-error parameter tuning, as in the study of Kocieniewski and Lipniacki (2013), with a reproducible, automated pipeline that integrates both quantitative and qualitative data. We showed how to formalize up/down results of case-control comparisons as Biological Property Specification Language (BPSL) statements, and we showed how to leverage BPSL statements in concert with conventional time-series data in not only model parameterization but also rigorous uncertainty quantification (UQ). We elaborated on the approach of Mitra et al. (2018), showing that this approach is grounded in likelihood-based inference. We also demonstrated both frequentist and Bayesian approaches to rigorous uncertainty quantification (UQ), which was missing in the original modeling study of Kocieniewski and Lipniacki (2013).

Our results illustrate several advantages of a PyBioNetFit-enabled workflow, including potential for straightforward reusability of qualitative observations, once formalized as BPSL statements, and reproducibility of complex workflows. PyBioNetFit job setup files consist of easily shared plain-text files capturing data, models, and algorithms in standardized formats. Our results also reinforce the value of qualitative data. These data can be used in model parameterization with UQ, in multiple ways. Looking ahead, this study serves as a template for enhancing model parameterization pipelines in contexts where qualitative data are available to complement (limited) quantitative data.

## Supplementary Material

Supplement 1

## Figures and Tables

**Table 1:** Original parameter value estimates of the MEK isoform model determined by Kocieniewski and Lipniacki (2013).

Parameter	Original Parameter Value	Parameter Description
c_1_L	2.0×10^−2^	c1 value after stimulation with ligand
c_2_	2.0×10^−7^	EGFR receptor dimerization dueligand
t_1_	1.0×10^2^	EGFR subunits transphosphorylation in EGFR dimer
d_3_	1.0×10^−3^	degradation of ligand bound dimer complexes
b_1_	4.0×10^−8^	association of phosphorylated receptor EGFR subunits with SOS1
n_1_	2.0×10^−3^	disassociation of EGFR receptor subunits from SOS1
b_2_	1.0×10^−5^	MEK1 homodimer formation
n_2_	1.0×10^−3^	MEK1 dimer dissociation
b_3_	1.0×10^−5^	MEK2 homodimer formation
n_3_	3.0×10^−2^	MEK2 homodimer dissociation
b_4_	1.0×10^−5^	MEK1 and MEK2 heterodimer formation
n_4_	1.0×10^−3^	MEK1 and MEK2 heterodimer dissociation
a_1_	1.5×10^−7^	activation of Ras by EGFR SOS1 complex (exchange of GDP for GTP)
i_1_	2.0×10^−2^	inactivation of RAS (hydrolysis of bound GTP to GDP)
a_2_	4.0×10^−8^	activation of RAF by RAS – GTP
i_2_	1.0×10^−2^	inactivation of RAF
p_1_	1.5×10^−7^	phosphorylation of MEK1 and MEK2 on the activation sites by RAF
u_1_	5.0×10^−3^	dephosphorylation of MEK1 and MEK2 on the activation sites
p_2_a	1.0×10^−6^	phosphorylation of ERK on the activation sites by MEK1
p_2_b	5.0×10^−6^	phosphorylation of ERK on the activation sites by MEK2
u_2_	2.0×10^−2^	dephosphorylation of ERK on the activation sites
p_3_	2.0×10^−2^	feedback phosphorylation of SOS1 by active ERK
u_3_	1.0×10^−3^	dephosphorylation of the SOS1 feedback site
p_4_	1.2×10^−9^	feedback phosphorylation of MEK1 by ERK
u_4_	2.0×10^−4^	dephosphorylation of the MEK1 feedback site (Thr292)
b_5_	4.0×10^−9^	PHP phosphatse binding to Thr292p of MEK1
n_5_	2.0×10^−4^	dissociation of PHP phosphatase from Thr292p of MEK1
u_5_	2.0×10^1^	dephosphorylation of MEK1 and MEK2 on the activation sites
*s*_1_ *	2.5	EGFR receptor subunit constitutive production
*d*_1_ *	5.0×10^−6^	EGFR receptor subunit constitutive degredation
*s*_2_ *	1.0	Sos1 constitutive production
*d*_2_ *	5.0×10^−6^	Sos1 constitutive degredation
*c*_1_ *	2.0×10^−2^	formation of EGFR receptor subunit – ligand complex **Starting Signal**
*c*1*_init_* *	0.0	*c*_1_ value after simulation with ligand
MEK1_fraction*	6.7×10^−1^	MEK1 fraction of total MEKs content
X*	5.0	MEK2 – MEK1 kinase activity ratio
EGFR_0_*	5.0×10^5^	initial EGFR level
SOS1_0_*	2.0×10^5^	initial Sos1 level
RAS_0_*	5.0×10^5^	initial RAS level
RAF_0_*	5.0×10^5^	initial RAF level
MEK_tot_0_*	2.0×10^5^	initial MEK1 and MEK2 total level
MEK1_0_*	1.34×10^5^	initial MEK1 level
MEK1_T292p_0_*	0.0	initial MEK1 – Thr292p level
MEK2_0_*	6.6×10^4^	initial MEK2 level
ERK_0_*	3.0×10^6^	initial combined ERK1 and ERK2 level
PHP_MEK_0_*	3.0×10^6^	initial PHP level

All original 46 parameters are shown, including a brief description of their function within the MEK isoform model. Parameters marked with a single asterisk (*) denote parameters that were fixed during PyBioNetFit’s automated parameterization of the model.

**Table 2:** Best-fit parameter estimates.

Parameter	Best-fit Value	Optimization Bounds [low, high]	Bayesian UQ Bounds [low, high]
c_1_L	7.4×10^−3^	[9.0×10^−4^, 4.0×10^−2^]	N/A
c_2_	9.3×10^−9^	[9.0×10^−9^, 4.0×10^−6^]	N/A
t_1_	9.9×10^1^	[99, 101]	N/A
d_3_	2.0×10^−3^	[8.0×10^−5^, 3.0×10^−2^]	[1.0×10^−5^, 1.0×10^−1^]
b_1_	2.4×10^−8^	[2.0×10^−9^, 6.0×10^−7^]	N/A
n_1_	6.5×10^−4^	[9.0×10^−5^, 4.0×10^−2^]	N/A
b_2_	4.2×10^−6^	[8.0×10^−7^, 3.0×10^−4^]	N/A
n_2_	2.6×10^−4^	[8.0×10^−5^, 3.0×10^−2^]	N/A
b_3_	9.9×10^−5^	[8.0×10^−7^, 3.0×10^−4^]	N/A
n_3_	8.0×10^−2^	[1.0×10^−3^, 5.0×10^−1^]	N/A
b_4_	2.9×10^−4^	[8.0×10^−7^, 3.0×10^−4^]	N/A
n_4_	3.5×10^−3^	[8.0×10^−5^, 3.0×10^−2^]	N/A
a_1_	2.3×10^−7^	[1.27×10^−8^, 1.29×10^−6^]	N/A
i_1_	3.9×10^−1^	[9.0×10^−4^, 4.0×10^−1^]	N/A
a_2_	3.3×10^−8^	[2.0×10^−9^, 6.0×10^−7^]	N/A
i_2_	2.7×10^−2^	[8.0×10^−4^, 3.0×10^−1^]	N/A
p_1_	3.5×10^−6^	[8.0×10^−9^, 4.0×10^−6^]	N/A
u_1_	9.5×10^−4^	[3.0×10^−4^, 7.0×10^−2^]	N/A
p_2_a	5.2×10^−7^	[8.0×10^−8^, 3.0×10^−5^]	N/A
p_2_b	2.3×10^−5^	[1.0×10^−6^, 5.0×10^−4^]	N/A
u_2_	6.7×10^−2^	[9.0×10^−4^, 4.0×10^−1^]	N/A
p_3_	1.1×10^−9^	[9.0×10^−11^, 4.0×10^−8^]	N/A
u_3_	2.6×10^−4^	[8.0×10^−5^, 3.0×10^−2^]	[1.0×10^−5^, 1.0×10^−1^]
p_4_	6.5×10^−10^	[9.0×10^−11^, 3.0×10^−8^]	N/A
u_4_	3.2×10^−4^	[9.0×10^−6^, 4.0×10^−3^]	N/A
b_5_	1.7×10^−8^	[2.0×10^−10^, 6.0×10^−8^]	N/A
n_5_	3.9×10^−3^	[9.0×10^−6^, 4.0×10^−3^]	N/A
u_5_	1.9×10^1^	[19, 21]	N/A
Scale_pEGFR_*	1.1×10^−4^	[3.0×10^−5^, 6.0×10^−5^]	[3.0×10^−5^, 6.0×10^−5^]
Scale_pERK_*	3.2×10^−6^	[2.0×10^−6^, 5.0×10^−6^]	[2.0×10^−6^, 5.0×10^−6^]
Scale_pSOS1_*	1.1×10^−4^	[9.0×10^−5^, 3.0^−4^]	[9.0×10^−5^, 3.0^−4^]
*sigma* **	1.1	N/A	[1.0×10^−1^, 1.0×10^1^]

This table lists the 31 parameters selected for model calibration using PyBioNetFit. Each row indicates the name of a parameter, the best-fit value obtained from a global fit, and parameter bounds used in optimization and Bayesian UQ (if applicable). Parameters marked with a single asterisk (*) are scaling factors added specifically for the WT model to allow model outputs to be compared to published measurements reported in arbitrary units (AU). Parameters with a double asterisk (**) are special hyper sampling parameters used only in Bayesian UQ simulations needed for PyBioNetFit’s Adaptive MCMC algorithm and not originally included in the model by Kocieniewski and Lipniacki (2013).

**Table 3.** Sampling convergence metrics.

	ESS_Bulk_	ESS_Tail_	$\hat{R}-1$
$d_{3}$	6160	8620	1.8×10^−4^
$u_{3}$	2150	2120	6.0×10^−4^

For the rate constants $d_{3}$ and $u_{3}$, the table reports the *bulk* and *tail* Effective Sample Size (ESS), as well as the quantity $\hat{R}-1$, in which $\hat{R}$ is a convergence diagnostic derived from the potential scale reduction factor and computed using the rstan R package. ESS_Bulk_ evaluates the effective number of independent samples for the central bulk of the posterior distribution, whereas ESS_Tail_ reflects the stability of sampling in the distribution tails. High ESS values across both metrics indicate efficient sampling with low autocorrelation. The $\hat{R}-1$ values are close to zero, suggesting convergence of the chains and well-mixed posterior estimates.

## Data Availability

The code, datasets, and job setup files used in this study can be found in the PyBioNetFit GitHub repository (https://github.com/lanl/PyBNF).
