# Supplementary material for: Using PyBioNetFit to Leverage Qualitative and Quantitative Data in Biological Model Parameterization and Uncertainty Quantification
Source: ArXiv. 2025 Aug 26:arXiv:2508.19420v1. Preprint. [Version 1] (PMC12407630)
Supplement: Supplement 1 [file NIHPP2508.19420v1-supplement-1.pdf]

11 Supplementary Material

The source code used in this study can be found in the PyBioNetFit GitHub repository (https://github.com/lanl/PyBNF). Within this repository, the datasets and job setup files used in this study for maximum likelihood estimation and Bayesian inference can be found here: https://github.com/lanl/PyBNF/tree/master/examples/Miller2025_MEK_Isoforms

Supplementary Tables and Figures

Constraint #	WT Model

C1	WT.MEK_pRDS at time=300 > WT.MEK_pRDS at time=1800
C2	WT.MEK_pRDS at time=1800 > WT.MEK_pRDS at time=3600
C3	WT.MEK_pRDS at time=300 > WT.MEK_pRDS at time=3600
C4	WT.MEK_pRDS at time=300 > KO.MEK_pRDS at time=300
C5	WT.MEK_pRDS at time=1800 < KO.MEK_pRDS at time=1800
C6	WT.MEK_pRDS at time=3600 < KO.MEK_pRDS at time=3600
C7	WT.MEK_pRDS at time=300 < N78G.MEK_pRDS at time=300
C8	WT.MEK_pRDS at time=1800 < N78G.MEK_pRDS at time=1800
C9	WT.MEK_pRDS at time=3600 < N78G.MEK_pRDS at time=3600
C10	WT.MEK_pRDS at time=300 < T292A.MEK_pRDS at time=300
C11	WT.MEK_pRDS at time=1800 < T292A.MEK_pRDS at time=1800
C12	WT.MEK_pRDS at time=3600 < T292A.MEK_pRDS at time=3600
C13	WT.MEK_pRDS at time=300 > T292D.MEK_pRDS at time=300
C14	WT.MEK_pRDS at time=1800 > T292D.MEK_pRDS at time=1800
C15	WT.MEK_pRDS at time=3600 > T292D.MEK_pRDS at time=3600
C16	WT.pERK1_2 at time=300 > WT.pERK1_2 at time=1800
C17	WT.pERK1_2 at time=1800 > WT.pERK1_2 at time=3600
C18	WT.pERK1_2_ at time=300 > WT.pERK1_2 at time=3600
C19	WT.pERK1_2 at time=300 > KO.pERK1_2 at time=300
C20	WT.pERK1_2 at time=1800 < KO.pERK1_2 at time=1800

C21	WT.pERK1_2 at time=3600 < KO.pERK1_2 at time=3600
C22	WT.pERK1_2 at time=300 > N78G.pERK1_2 at time=300
C23	WT.pERK1_2 at time=1800 < N78G.pERK1_2 at time=1800
C24	WT.pERK1_2 at time=3600 < N78G.pERK1_2 at time=3600
C25	WT.pERK1_2 at time=300 < T292A.pERK1_2 at time=300
C26	WT.pERK1_2 at time=1800 < T292A.pERK1_2 at time=1800
C27	WT.pERK1_2 at time=3600 < T292A.pERK1_2 at time=3600
C28	WT.pERK1_2 at time=300 > T292D.pERK1_2 at time=300
C29	WT.pERK1_2 at time=1800 > T292D.pERK1_2 at time=1800
C30	WT.pERK1_2 at time=3600 > T292D.pERK1_2 at time=3600

Supplemental Table 1 (WT constraint glossary):
This table provides a glossary for the constraint labels (C1–C30) used in Supplemental Table 6 for the WT model. Each entry is a BPSL statement that formalizes a qualitative observation. Time points in the statements are given in seconds.

Constraint #	KO Model
C1	KO.MEK_pRDS at time=300 > KO.MEK_pRDS at time=1800

C2	KO.MEK_pRDS at time=300 > KO.MEK_pRDS at time=3600
C3	KO.MEK_pRDS at time=1800 > KO.MEK_pRDS at time=3600
C4	KO.MEK_pRDS at time=300 < N78G.MEK_pRDS at time=300
C5	KO.MEK_pRDS at time=1800 < N78G.MEK_pRDS at time=1800
C6	KO.MEK_pRDS at time=3600 > N78G.MEK_pRDS at time=3600
C7	KO.MEK_pRDS at time=300 < T292A.MEK_pRDS at time=300
C8	KO.MEK_pRDS at time=1800 < T292A.MEK_pRDS at time=1800
C9	KO.MEK_pRDS at time=3600 < T292A.MEK_pRDS at time=3600
C10	KO.MEK_pRDS at time=300 > T292D.MEK_pRDS at time=300
C11	KO.MEK_pRDS at time=1800 > T292D.MEK_pRDS at time=1800
C12	KO.MEK_pRDS at time=3600 > T292D.MEK_pRDS at time=3600
C13	KO.pERK1_2 at time=300 > KO.pERK1_2at time=1800
C14	KO.pERK1_2 at time=1800 > KO.pERK1_2 at time=3600
C15	KO.pERK1_2 at time=300 > KO.pERK1_2 at time=3600
C16	KO.pERK1_2 at time=300 < N78G.pERK1_2 at time=300
C17	KO.pERK1_2 at time=1800 < N78G.pERK1_2 at time=1800
C18	KO.pERK1_2 at time=3600 < N78G.pERK1_2 at time=3600
C19	KO.pERK1_2 at time=300 < T292A.pERK1_2 at time=300
C20	KO.pERK1_2 at time=1800 < T292A.pERK1_2 at time=1800
C21	KO.pERK1_2 at time=3600 < T292A.pERK1_2 at time=3600

C22	KO.pERK1_2 at time=300 > T292D.pERK1_2 at time=300
C23	KO.pERK1_2 at time=1800 > T292D.pERK1_2 at time=1800
C24	KO.pERK1_2 at time=3600 > T292D.pERK1_2 at time=3600

Supplemental Table 2 (KO constraint glossary):
This table provides a glossary for the constraint labels (C1–C24) used in Supplemental Table 6 for the KO model. Each entry is a BPSL statement that formalizes a qualitative observation. Time points in the statements are given in seconds.

\

Constraint #	N78G Model
C1	N78G.MEK_pRDS at time=300 > N78G.MEK_pRDS at time=1800
C2	N78G.MEK_pRDS at time=1800 > N78G.MEK_pRDS at time=3600

C3	N78G.MEK_pRDS at time=300 > N78G.MEK_pRDS at time=3600
C4	N78G.MEK_pRDS at time=300 < T292A.MEK_pRDS at time=300
C5	N78G.MEK_pRDS at time=1800 < T292A.MEK_pRDS at time=1800
C6	N78G.MEK_pRDS at time=3600 < T292A.MEK_pRDS at time=3600
C7	N78G.MEK_pRDS at time=300 > T292D.MEK_pRDS at time=300
C8	N78G.MEK_pRDS at time=1800 > T292D.MEK_pRDS at time=1800
C9	N78G.MEK_pRDS at time=3600 > T292D.MEK_pRDS at time=3600
C10	N78G.pERK1_2 at time=300 > N78G.pERK1_2 at time=1800
C11	N78G.pERK1_2 at time=1800 > N78G.pERK1_2 at time=3600
C12	N78G.pERK1_2 at time=300 > N78G.pERK1_2 at time=3600
C13	N78G.pERK1_2 at time=300 < T292A.pERK1_2 at time=300
C14	N78G.pERK1_2 at time=1800 < T292A.pERK1_2 at time=1800
C15	N78G.pERK1_2 at time=3600 < T292A.pERK1_2 at time=3600
C16	N78G.pERK1_2 at time=300 > T292D.pERK1_2 at time=300
C17	N78G.pERK1_2 at time=1800 > T292D.pERK1_2 at time=1800
C18	N78G.pERK1_2 at time=3600 > T292D.pERK1_2 at time=3600

Supplemental Table 3 (N78G constraint glossary):
This table provides a glossary for the constraint labels (C1–C18) used in Supplemental Table 6 for the N78G model. Each entry is a BPSL statement that formalizes a qualitative observation. Time points in the statements are given in seconds.

Constraint #	T292A Model
C1	T292A.MEK_pRDS at time=300 > T292A.MEK_pRDS at time=1800
C2	T292A.MEK_pRDS at time=300 > T292A.MEK_pRDS at time=3600
C3	T292A.MEK_pRDS at time=1800 > T292A.MEK_pRDS at time=3600
C4	T292A.MEK_pRDS at time=300 > T292D.MEK_pRDS at time=300

C5	T292A.MEK_pRDS at time=1800 > T292D.MEK_pRDS at time=1800
C6	T292A.MEK_pRDS at time=3600 > T292D.MEK_pRDS at time=3600
C7	T292A.pERK1_2 at time=300 > T292A.pERK1_2 at time=1800
C8	T292A.pERK1_2 at time=1800 > T292A.pERK1_2 at time=3600
C9	T292A.pERK1_2 at time=300 > T292A.pERK1_2 at time=3600
C10	T292A.pERK1_2 at time=300 > T292D.pERK1_2 at time=300
C11	T292A.pERK1_2 at time=1800 > T292D.pERK1_2 at time=1800
C12	T292A.pERK1_2 at time=3600 > T292D.pERK1_2 at time=3600

Supplemental Table 4 (T292A constraint glossary):
This table provides a glossary for the constraint labels (C1–C12) used in Supplemental Table 6 for the T292A model. Each entry is a BPSL statement that formalizes a qualitative observation. Time points in the statements are given in seconds.

Constraint #	T292D Model
C1	T292D.MEK_pRDS at time=300 > T292D.MEK_pRDS at time=1800
C2	T292D.MEK_pRDS at time=300 > T292D.MEK_pRDS at time=3600
C3	T292D.MEK_pRDS at time=1800 > T292D.MEK_pRDS at time=3600
C4	T292D.pERK1_2 at time=300 > T292D.pERK1_2 at time=1800
C5	T292D.pERK1_2 at time=1800 > T292D.pERK1_2 at time=3600

C6	T292D.pERK1_2 at time=300 > T292D.pERK1_2 at time=3600

Supplemental Table 5 (T929D constraint glossary):
This table provides a glossary for the constraint labels (C1–C30) used in Supplemental Table 6 for the T292D model. Each entry is a BPSL statement that formalizes a qualitative observation. Time points in the statements are given in seconds.

Constraint #	WT Model	KO Model	N78G Model	T292A Model	T292D Model
C1	100	100	100	100	100
C2	100	100	100	100	100
C3	100	100	100	100	100
C4	100	100	100	100	100
C5	100	100	100	100	0
C6	100	0.2	100	100	100

C7	100	100	100	100	--
C8	100	100	100	100	--
C9	100	100	100	100	--
C10	100	0	100	100	--
C11	100	100	100	100	--
C12	100	100	100	100	--
C13	100	100	100	--	--
C14	100	100	100	--	--
C15	99.8	100	100	--	--
C16	100	0	100	--	--
C17	100	0	100	--	--
C18	100	0	100	--	--
C19	0	0	--	--	--
C20	100	0	--	--	--
C21	100	0	--	--	--
C22	0	100	--	--	--
C23	100	100	--	--	--
C24	100	100	--	--	--
C25	100	--	--	--	--
C26	100	--	--	--	--

C27	100	--	--	--	--
C28	100	--	--	--	--
C29	100	--	--	--	--
C30	100	--	--	--	--

Supplemental Table 6: Percentage of accepted MCMC samples that satisfy the indicated constraint. A total of 90 constraints on predicted system behavior were defined using BPSL. Each entry is the percent of sampled parameter values yielding consistency with the indicated constraint. An entry of 100% indicates that a constraint was satisfied for sampled parameter sets, whereas an entry of 0% indicates that the constraint was never satisfied. Constraints are labeled C1–C30 and listed in the same top-down order as in each model's corresponding PROP file. Columns indicate individual models (e.g., the WT model), and rows indicate specific constraints. An entry of "--" indicates that a constraint was not applicable. See Supplemental Tables 1–5 for the BPSL statements that define the constraints.

	Original Parameterization	PyBioNetFit Parameterization
Objective Funtion Score	40.0	15.0

Supplemental Table 7: Overall objective function score for both Kocieniewski and Lipniacki's (2013) parameterization and PyBioNetFit's parameterization. Objective function scores were calculated using PyBioNetFit's Sum of Squares objective function and Differential Evolution fitting algorithm. Lower scores indicate better fits.

**Time (s)**	**Species**	**Experimental Data (AU)**	**RMSE (PyBioNetFit)**	**RMSE (Original)**
**0**	pSOS1	0.0	0.0	0.0
**0**	pEGFR	1.5	1.5	1.5
**0**	pERK	0.9	0.9	0.9
**300**	pSOS1	4.2	1.776	1.846

**300**	pEGFR	10.0	0.105	0.213
**300**	pERK	9.9	0.496	0.028
**600**	pSOS1	9.5	0.539	0.39
**600**	pEGFR	9.6	0.277	1.879
**600**	pERK	9.0	0.245	0.624
**900**	pSOS1	10.0	0.536	0.038
**900**	pEGFR	6.1	0.61	0.258
**900**	pERK	7.9	0.979	1.546
**1800**	pSOS1	9.5	0.251	0.977
**1800**	pEGFR	2.9	0.836	0.429
**1800**	pERK	5.0	0.391	2.368
**3600**	pSOS1	6.9	0.145	2.809
**3600**	pEGFR	1.9	1.579	1.421
**3600**	pERK	3.5	2.984	3.029

Supplemental Table 8: RMSE values for PyBioNetFit outputs vs original parameterization outputs at each time point for the data in the WT.exp file, available in the supplemental setup files in the LANL GitHub. Experimental AU data are compared to model outputs for each species (pSOS1, pEGFR, pERK), at six experimental sampling times, across both PyBioNetFit's parameterization and the original parameterization. The statistic shown is Root Mean Standard Error, highlighting the distance each model's parameterization output from the experimental data points. Lower RMSE scores is associated with a better fit to the data.

Parameterization	Global RMSE
**PyBioNetFit**	1.076
**Original**	1.47

Supplemental Table 9: Global RMSE scores for each model parameterization's output, considering all time points and experimental data points across all 3 species (Sos1, EGFR, ERK) as seen in Supplemental Table 8. Lower RMSE scores indicate a better fit to the experimental data.

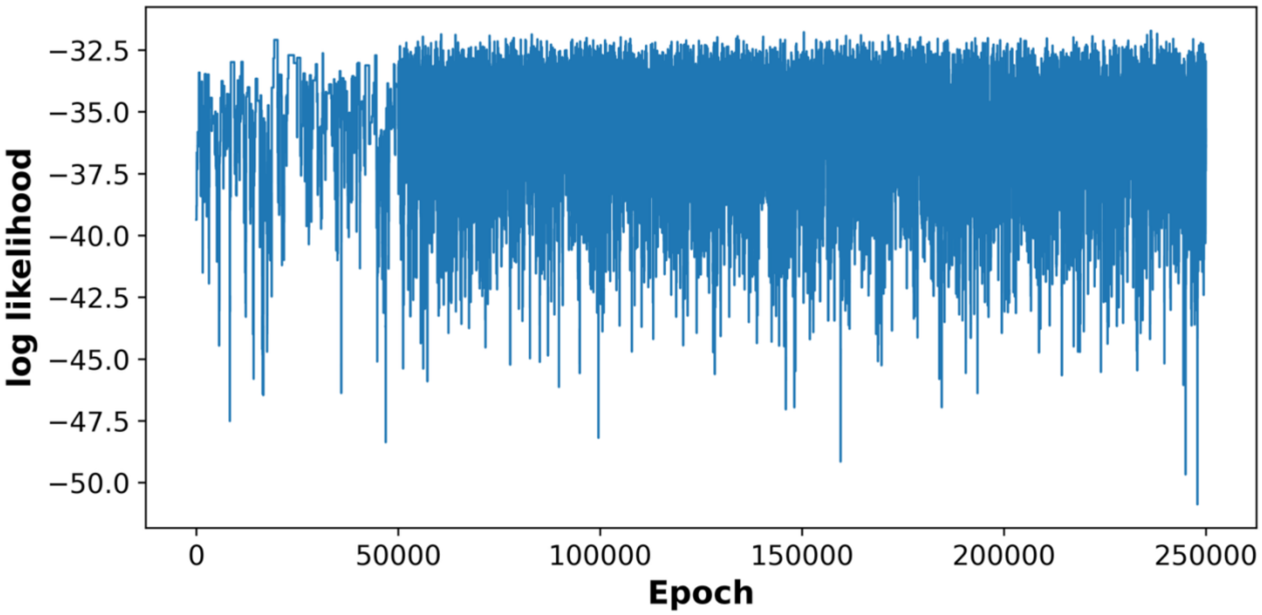

Supplementary Figure 1: Trace plot of log-likelihood values across 250,000 production iterations of MCMC sampling.

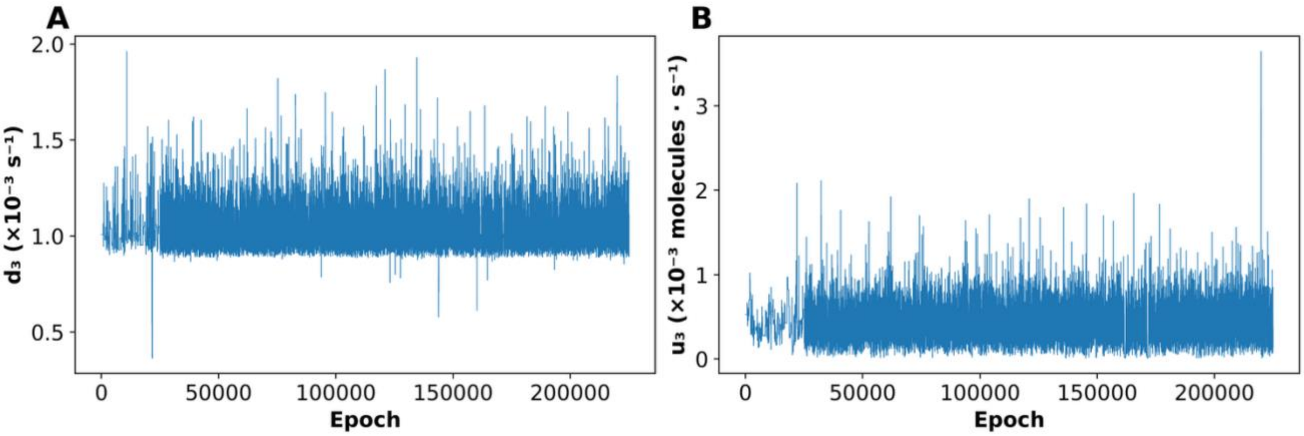

Supplementary Figure 2: Trace plots for each of two parameters across 250,000 iterations of production sampling. Panel A shows the trace plot for d_3, and panel B shows the trace plot for u_3.

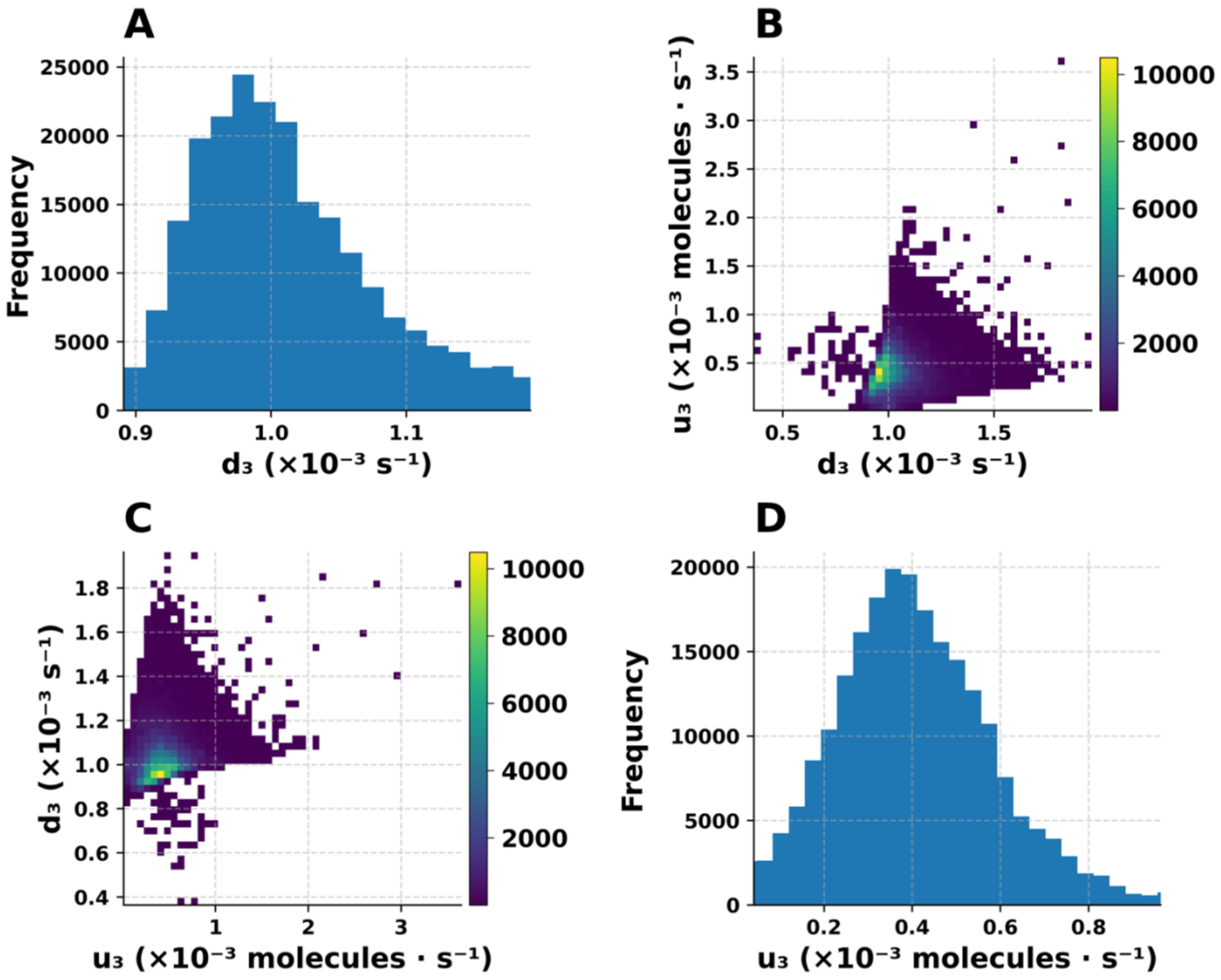

Supplementary Figure 3: Pairs plots of posterior samples for the rate constants d_3 and u_3. (A) Marginal posterior for d_3. (D) Marginal posterior for u_3. (B, C) Joint posterior density, i.e., the continuous two-dimensional density estimate of the posterior over (d_3, u_3). Colors indicate relative sample density, from white (lowest) to yellow (highest).

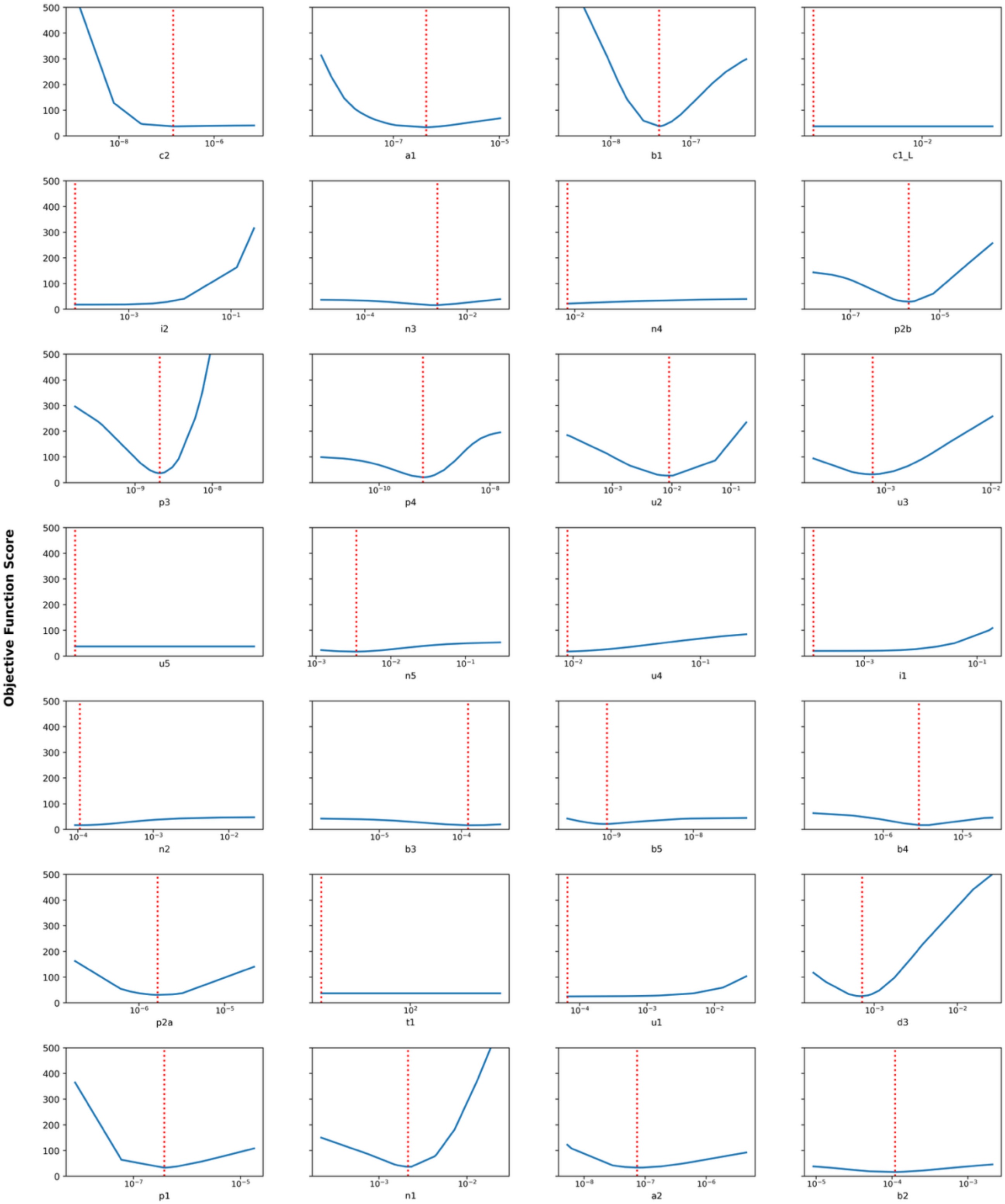

Supplementary Figure 4: A profile likelihood table of all 28 parameters used for parameterization with PyBioNetFit. The x axis for each plot shows what parameter value corresponds to which objective function score on the y axis. Vertical, red-dotted lines indicate the maximum likelihood estimate for that parameter. Parameters that are identifiable should have curved, bell-shaped, lines

(blue lines) that decrease in objective function score until they reach their maximum likelihood estimates (red-dotted lines). Parameters that are not identifiable appear flat.
